# The challenge of unprecedented floods and droughts in risk management

**DOI:** 10.1038/s41586-022-04917-5

**Published:** 2022-08-03

**Authors:** Heidi Kreibich, Anne F. Van Loon, Kai Schröter, Philip J. Ward, Maurizio Mazzoleni, Nivedita Sairam, Guta Wakbulcho Abeshu, Svetlana Agafonova, Amir AghaKouchak, Hafzullah Aksoy, Camila Alvarez-Garreton, Blanca Aznar, Laila Balkhi, Marlies H. Barendrecht, Sylvain Biancamaria, Liduin Bos-Burgering, Chris Bradley, Yus Budiyono, Wouter Buytaert, Lucinda Capewell, Hayley Carlson, Yonca Cavus, Anaïs Couasnon, Gemma Coxon, Ioannis Daliakopoulos, Marleen C. de Ruiter, Claire Delus, Mathilde Erfurt, Giuseppe Esposito, Didier François, Frédéric Frappart, Jim Freer, Natalia Frolova, Animesh K. Gain, Manolis Grillakis, Jordi Oriol Grima, Diego A. Guzmán, Laurie S. Huning, Monica Ionita, Maxim Kharlamov, Dao Nguyen Khoi, Natalie Kieboom, Maria Kireeva, Aristeidis Koutroulis, Waldo Lavado-Casimiro, Hong-Yi Li, María Carmen LLasat, David Macdonald, Johanna Mård, Hannah Mathew-Richards, Andrew McKenzie, Alfonso Mejia, Eduardo Mario Mendiondo, Marjolein Mens, Shifteh Mobini, Guilherme Samprogna Mohor, Viorica Nagavciuc, Thanh Ngo-Duc, Thi Thao Nguyen Huynh, Pham Thi Thao Nhi, Olga Petrucci, Hong Quan Nguyen, Pere Quintana-Seguí, Saman Razavi, Elena Ridolfi, Jannik Riegel, Md Shibly Sadik, Elisa Savelli, Alexey Sazonov, Sanjib Sharma, Johanna Sörensen, Felipe Augusto Arguello Souza, Kerstin Stahl, Max Steinhausen, Michael Stoelzle, Wiwiana Szalińska, Qiuhong Tang, Fuqiang Tian, Tamara Tokarczyk, Carolina Tovar, Thi Van Thu Tran, Marjolein H. J. Van Huijgevoort, Michelle T. H. van Vliet, Sergiy Vorogushyn, Thorsten Wagener, Yueling Wang, Doris E. Wendt, Elliot Wickham, Long Yang, Mauricio Zambrano-Bigiarini, Günter Blöschl, Giuliano Di Baldassarre

**Affiliations:** 1 GFZ German Research Centre for Geosciences, Section Hydrology, Potsdam, Germany; 2 Institute for Environmental Studies, Vrije Universiteit Amsterdam, Amsterdam, the Netherlands; 3 Leichtweiss Institute for Hydraulic Engineering and Water Resources, Division of Hydrology and River basin management, Technische Universität Braunschweig, Braunschweig, Germany; 4 Department of Civil and Environmental Engineering, University of Houston, Houston, TX USA; 5 Lomonosov Moscow State University, Moscow, Russia; 6 University of California, Irvine, CA USA; 7 Department of Civil Engineering, Istanbul Technical University, Istanbul, Turkey; 8 Center for Climate and Resilience Research, Santiago, Chile; 9 Department of Civil Engineering, Universidad de La Frontera, Temuco, Chile; 10 Operations Department, Barcelona Cicle de l’Aigua S.A, Barcelona, Spain; 11 Global Institute for Water Security, University of Saskatchewan, Saskatoon, Saskatchewan Canada; 12 LEGOS, Université de Toulouse, CNES, CNRS, IRD, UPS, Toulouse, France; 13 Department of Groundwater Management, Deltares, Delft, the Netherlands; 14 School of Geography, Earth and Environmental Sciences, University of Birmingham, Birmingham, UK; 15 Agency for the Assessment and Application of Technology, Jakarta, Indonesia; 16 Department of Civil and Environmental Engineering, Imperial College London, London, UK; 17 Department of Civil Engineering, Beykent University, Istanbul, Turkey; 18 Graduate School, Istanbul Technical University, Istanbul, Turkey; 19 Faculty of Environment and Natural Resources, University of Freiburg, Freiburg, Germany; 20 Geographical Sciences, University of Bristol, Bristol, UK; 21 Cabot Institute, University of Bristol, Bristol, UK; 22 Department of Agriculture, Hellenic Mediterranean University, Iraklio, Greece; 23 Université de Lorraine, LOTERR, Metz, France; 24 CNR-IRPI, Research Institute for Geo-Hydrological Protection, Cosenza, Italy; 25 INRAE, Bordeaux Sciences Agro, UMR ISPA, Villenave dʼOrnon, France; 26 University of Saskatchewan, Centre for Hydrology, Canmore, Alberta Canada; 27 Environmental Policy and Planning Group, Department of Urban Studies and Planning, Massachusetts Institute of Technology, Cambridge, MA USA; 28 Department of Economics, Ca’ Foscari University of Venice, Venice, Italy; 29 Lab of Geophysical-Remote Sensing & Archaeo-environment, Institute for Mediterranean Studies, Foundation for Research and Technology Hellas, Rethymno, Greece; 30 Pontificia Bolivariana University, Faculty of Civil Engineering, Bucaramanga, Colombia; 31 California State University, Long Beach, CA USA; 32 Alfred Wegener Institute Helmholtz Center for Polar and Marine Research, Palaeoclimate Dynamics Group, Bremerhaven, Germany; 33 Emil Racovita Institute of Speleology, Romanian Academy, Cluj-Napoca, Romania; 34 Forest Biometrics Laboratory, Faculty of Forestry, Ștefan cel Mare University, Suceava, Romania; 35 Water Problem Institute Russian Academy of Science, Moscow, Russia; 36 Faculty of Environment, University of Science, Ho Chi Minh City, Vietnam; 37 Environment Agency, Bristol, UK; 38 School of Chemical and Environmental Engineering, Technical University of Crete, Chania, Greece; 39 Servicio Nacional de Meteorología e Hidrología del Perú, Lima, Peru; 40 Department of Applied Physics, University of Barcelona, Barcelona, Spain; 41 Water Research Institute, University of Barcelona, Barcelona, Spain; 42 British Geological Survey, Wallingford, UK; 43 Centre of Natural Hazards and Disaster Science, Uppsala, Sweden; 44 Department of Earth Sciences, Uppsala University, Uppsala, Sweden; 45 Civil and Environmental Engineering, The Pennsylvania State University, State College, PA USA; 46 Escola de Engenharia de Sao Carlos, University of São Paulo, São Paulo, Brasil; 47 Department of Water Resources & Delta Management, Deltares, Delft, the Netherlands; 48 Trelleborg municipality, Trelleborg, Sweden; 49 Department of Water Resources Engineering, Lund University, Lund, Sweden; 50 University of Potsdam, Institute of Environmental Science and Geography, Potsdam, Germany; 51 University of Science and Technology of Hanoi, Vietnam Academy of Science and Technology, Hanoi, Vietnam; 52 Institute for Environment and Resources, Vietnam National University Ho Chi Minh City, Ho Chi Minh City, Vietnam; 53 Institute for Circular Economy Development, Vietnam National University Ho Chi Minh City, Ho Chi Minh City, Vietnam; 54 Observatori de l’Ebre, Ramon Llull University – CSIC, Roquetes, Spain; 55 School of Environment and Sustainability, University of Saskatchewan, Saskatoon, Saskatchewan Canada; 56 Department of Civil, Geological and Environmental Engineering, University of Saskatchewan, Saskatoon, Saskatchewan Canada; 57 Dipartimento di Ingegneria Civile, Edile e Ambientale, Sapienza Università di Roma, Rome, Italy; 58 University of Applied Sciences, Magdeburg, Germany; 59 Center for Environmental and Geographic Information Services, Dhaka, Bangladesh; 60 Earth and Environmental Systems Institute, The Pennsylvania State University, State College, PA USA; 61 Institute of Meteorology and Water Management National Research Institute, Warsaw, Poland; 62 Key Laboratory of Water Cycle and Related Land Surface Processes, Institute of Geographical Sciences and Natural Resources Research, Chinese Academy of Sciences, Beijing, China; 63 Department of Hydraulic Engineering, Tsinghua University, Beijing, China; 64 Royal Botanical Gardens Kew, London, UK; 65 KWR Water Research Institute, Nieuwegein, the Netherlands; 66 Department of Physical Geography, Utrecht University, Utrecht, the Netherlands; 67 Civil Engineering, University of Bristol, Bristol, UK; 68 School of Natural Resources, University of Nebraska-Lincoln, Lincoln, NE USA; 69 School of Geography and Ocean Science, Nanjing University, Nanjing, China; 70 Institute of Hydraulic Engineering and Water Resources Management, Technische Universität Wien, Vienna, Austria; 71 Department of Integrated Water Systems and Governance, IHE Delft, Delft, the Netherlands

**Keywords:** Hydrology, Natural hazards

## Abstract

Risk management has reduced vulnerability to floods and droughts globally^1,2^, yet their impacts are still increasing^3^. An improved understanding of the causes of changing impacts is therefore needed, but has been hampered by a lack of empirical data^4,5^. On the basis of a global dataset of 45 pairs of events that occurred within the same area, we show that risk management generally reduces the impacts of floods and droughts but faces difficulties in reducing the impacts of unprecedented events of a magnitude not previously experienced. If the second event was much more hazardous than the first, its impact was almost always higher. This is because management was not designed to deal with such extreme events: for example, they exceeded the design levels of levees and reservoirs. In two success stories, the impact of the second, more hazardous, event was lower, as a result of improved risk management governance and high investment in integrated management. The observed difficulty of managing unprecedented events is alarming, given that more extreme hydrological events are projected owing to climate change^3^.

## Abstract (web summary)

Unprecedented floods and droughts bring new challenges for risk reduction, as is clear from this analysis of the drivers of changing impacts in many cases worldwide, with implications for efficient governance and investment in integrated management.

## Main

Observed decreasing trends in the vulnerability to floods and droughts, owing to effective risk management, are encouraging^1^. Globally, human and economic vulnerability dropped by approximately 6.5- and 5-fold, respectively, between the periods 1980–1989 and 2007–2016 (ref. ^2^). However, the impacts of floods and droughts are still severe and increasing in many parts of the world^6^. Climate change will probably lead to a further increase in their impacts owing to projected increases in the frequency and severity of floods and droughts^3^. The economic damage of floods is projected to double globally^7^ and that of droughts to triple in Europe^8^, for a mean temperature increase of 2 °C.

The purpose of risk management is to reduce the impact of events through modification of the hazard, exposure and/or vulnerability: according to United Nations (UN) terminology^9^, disaster risk management is the application of disaster risk reduction policies and strategies to prevent new disaster risk, reduce existing disaster risk and manage residual risk, contributing to the strengthening of resilience against, and reduction of, disaster losses. Hazard is a process, phenomenon or human activity that may cause loss of life, injury or other health impacts, property damage, social and economic disruption or environmental degradation; exposure is the situation of people, infrastructure, housing, production capacities and other tangible human assets located in hazard-prone areas; and vulnerability is the conditions determined by physical, social, economic and environmental factors or processes^10–13^ that increase the susceptibility of an individual, a community, assets or systems to the impacts of hazards. To be effective, risk management needs to be based on a sound understanding of these controlling risk drivers^14,15^. Past studies have identified increasing exposure as a primary driver of increasing impacts^3,4^, and vulnerability reduction has been identified as key for reduction of impacts^16,17^. However, ascertaining the combined effect of the drivers and the overall effectiveness of risk management has been hampered by a lack of empirical data^4,5^.

**Fig. 1:**
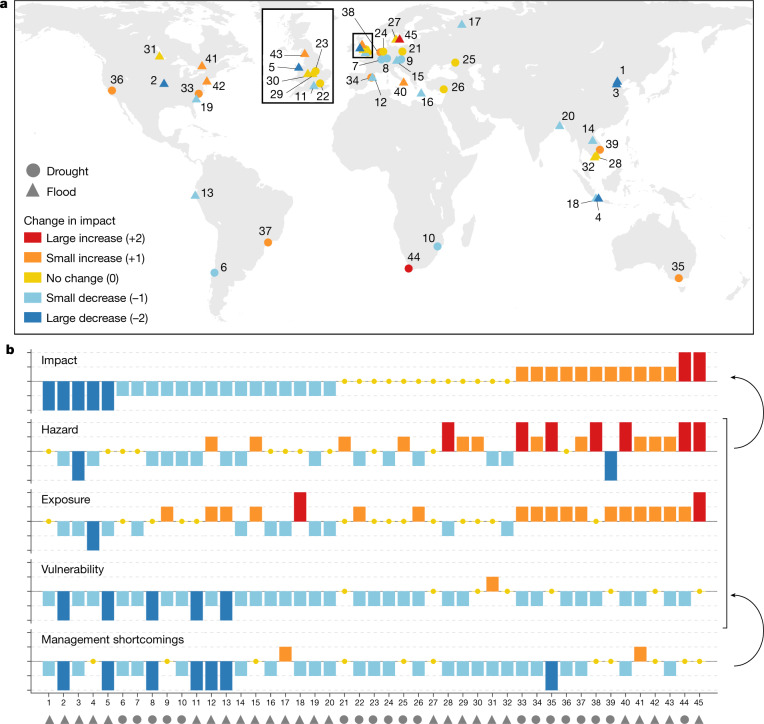
Location of flood and drought paired events coloured according to changes in impact and their indicators of change. **a**, Location of flood and drought paired events (*n* = 45). Numbers are paired-event IDs. **b**, Indicators of change, sorted by impact change. Impact is considered to be controlled by hazard, exposure and vulnerability, which are exacerbated by risk management shortcomings. Maps of the paired events coloured according to drivers and management shortcomings are shown in Extended Data Fig. 1. Source data

Here we analyse a new dataset of 45 pairs of flood or drought events that occurred in the same area on average 16 years apart (hereinafter referred to as paired events). The data comprise 26 flood and 19 drought paired events across different socioeconomic and hydroclimatic contexts from all continents (Fig. 1a). We analyse floods and droughts together, because of the similarity of some of the management methods (for example, warning systems, water reservoir infrastructure), the potential for trade-offs in risk reduction between floods and droughts and therefore value for the management communities to learn from each other^18^. The impact, quantified by direct (fatalities, monetary damage), indirect (for example, disruption of traffic or tourism) and intangible impacts (for example, impact on human health or cultural heritage), is considered to be controlled by three drivers: hazard, exposure and vulnerability^3^. These drivers are quantified using a large range of different indices—for example, the standardized precipitation index, the number of houses in the affected area and risk awareness, respectively (Supplementary Table 1). These three drivers are considered to be exacerbated by management shortcomings. Hazard may be exacerbated by problems with water management infrastructure such as levees or reservoirs^19^. Exposure and vulnerability may be worsened by suboptimal implementation of non-structural measures such as risk-aware regional planning^20^ or early warning^21^, respectively. We analyse management shortcomings and their effect on the three drivers explicitly, as this is the point at which improvements can start—for example, by the introduction of better strategies and policies. Data availability understandably varies among the paired events, and this can introduce inconsistency and subjectivity. The analyses are therefore based on indicators of change, to account for differences between paired events in respect of measured variables, data quality and uncertainty. These indicators of change represent the differences between the first event (baseline) and the second, categorized as large decreases/increases (−2/+2), small decreases/increases (−1/+1) and no change (0) (Supplementary Table 2). To minimize the subjectivity and uncertainty of indicator assignment, a quality assurance protocol is implemented and indicators of change with sub-indicators are used.

The majority of paired events show decreases in management shortcomings (71% of paired events; Fig. 1b), which reflects that societies tend to learn from extreme events^22^. Most cases also show a decrease in vulnerability (80% of paired events) as societies typically reduce their vulnerability after the first event of a pair^21^. The five paired events with a large decrease in impact (dark blue, top left in Fig. 1b) are associated with decreases or no change of all three drivers.

## Drivers of changes in impact

**Fig. 2:**
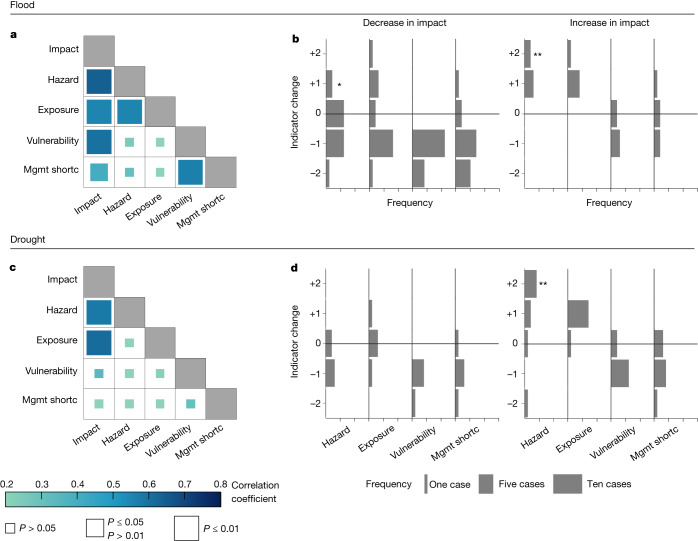
Correlation matrix and histograms of indicators of change. **a**, **c**, Correlation matrix of indicators of change for flood (**a**) and drought (**c**) paired events. Colours of squares indicate Spearman’s rank correlation coefficients and their size, the *P* value. **b**, **d**,Histograms of indicators of change of flood (**b**) and drought (**d**) stratified by decrease (*n* = 15 and *n* = 5 paired events for flood and drought, respectively) and increase (*n* = 5 and *n* = 8 paired events, respectively) in impact. The asterisk denotes the success stories of Box 1; double asterisks denote pairs for which the second event was much more hazardous than the first (that is, 'unprecedented'). Mgmt shortc, management shortcomings. Source data

Changes in flood impacts are significantly and positively correlated with changes in hazard (*r* = 0.64, *P* ≤ 0.01), exposure (*r* = 0.55, *P* ≤ 0.01) and vulnerability (*r* = 0.60, *P* ≤ 0.01) (Fig. 2a), which is in line with risk theory^3^. Although a previous analysis of eight case studies^21^ identified vulnerability as a key to reduction of flood impacts, this new, more comprehensive, dataset suggests that changes in hazard, exposure and vulnerability are equally important, given that they correlate equally strongly with changes in flood impact. Changes in drought impacts are significantly correlated with changes in hazard and exposure, but not with changes in vulnerability (Fig. 2c). This suggests that changes in vulnerability have been less successful in reducing drought impact than flood impact, which is also consistent with those event pairs for which only vulnerability changed (Extended Data Table 1). However, quantification of the contribution of individual drivers is difficult with this empirical approach because there are only a limited number of cases in which only one driver changed. There are three cases in which only vulnerability changed between events, two cases in which only hazard changed and no case in which only exposure changed (Extended Data Table 1). Additionally, paired events without a change in hazard (0) are analysed in more detail to better understand the role of exposure and vulnerability (Extended Data Fig. 2). In all these paired events, a reduction in impact was associated with a reduction in vulnerability, highlighting the importance of vulnerability. In five of these eight cases with a decrease in impact there was also a decrease in exposure, whereas in one case (floods in Jakarta, Indonesia in 2002 and 2007 (ID 18)) there was a large increase in exposure. In the paired event of droughts in California, United States (1987–1992 and 2011–2016, ID 36) an increase in exposure and a reduction in vulnerability increased impact, which points to the more important role of exposure in comparison with vulnerability in this drought case (Extended Data Fig. 2).

Generally the changes in drivers are not significantly correlated with each other, with the exception of hazard and exposure in the case of floods (*r* = 0.55, *P* ≤ 0.01) (Fig. 2a). This finding may be explained by the influence of hazard on the size of the inundation area, and thus on the numbers of people and assets affected, which represent exposure.

The sensitivity analysis suggests that the correlation pattern is robust, as visualized by the colours in Extended Data Fig. 3. The pattern of *P* values is also robust for flood cases, although these become less significant for drought because of the smaller sample size (Extended Data Fig. 3).

We split the paired events into groups of decreasing and increasing impact to evaluate their drivers separately (Fig. 2b,d). Overall, the pattern is similar for floods and droughts. Most flood and drought pairs with decreasing impact show either a decrease in hazard (ten pairs, 50%) or no change (eight pairs, 40%). Exceptions are two flood pairs that are success stories of decreased impact despite an increase in hazard, as detailed in Box 1. The change in exposure of the pairs with decreased impacts (Fig. 2b,d) ranges from a large decrease to a large increase, whereas vulnerability always decreased. All cases with a large decrease in vulnerability (−2) are associated with a decrease in impacts. Overall, the pattern suggests that a decrease in impacts is mainly caused by a combination of lower hazard and vulnerability, despite an increase in exposure in 25% of cases.

The role of hazard and vulnerability in impact reduction can be exemplified by the pair of riverine floods in Jakarta, Indonesia (ID 4 in Fig. 1). The 2007 event had a flood return period of 50 years, whereas it was 30 years for the 2013 event^23^ (that is, the hazard of the second event was smaller). Vulnerability had also decreased as a result of improved preparedness resulting from a flood risk mapping initiative and capacity building programmes implemented after the first flood, to improve citizens' emergency response, as well as by an improvement in official emergency management by establishment of the National Disaster Management Agency in 2008. Additionally, exposure was substantially reduced. Whilst the first flood caused 79 fatalities and direct damage of €1.3 billion, the second event caused 38 fatalities and €0.76 billion of direct damage.

Another example is a pair of Central European droughts (ID 9). During the 2003 event, the minimum 3-month Standardized Precipitation Evapotranspiration Index was −1.62 whereas in 2015 it was −1.18—that is, the hazard of the second event was smaller^24^. The vulnerability was also lower in the second event, because the first event had raised public awareness and triggered an improvement in institutional planning. For instance, the European Commission technical guidance on drought management plans^25^ was implemented. Many reservoirs were kept filled until the beginning of summer 2015, which alleviated water shortages for various sectors and, in some cities (for example, Bratislava and Bucharest), water was supplied from tanks^26^. Additionally, water use and abstraction restrictions were implemented for non-priority uses including irrigation^26^. The impact was reduced from €17.1 billion to €2.2 billion, despite an increase in exposure because of the larger drought extent affecting almost all of Europe in 2013.

Most flood and drought pairs with an increase in impact also show a larger hazard (11 cases, 85%; Fig. 2b,d). For six of these paired events (46%), the second event was much more hazardous than the first (hazard indicator-of-change +2), whereas this was never the case for the pairs with decreasing impact. Of those pairs with an increase in impact, 12 (92%) show an increase in exposure and nine (69%) show a small decrease in vulnerability (vulnerability indicator-of-change −1). Overall, the pattern suggests that the increase in impact is mainly caused by a combination of higher hazard and exposure, which is not compensated by a small decrease in vulnerability.

The role of hazard and exposure in increasing impact is illustrated by a pair of pluvial floods in Corigliano-Rossano City, Calabria, Italy (ID 40). This 2015 event was much more hazardous (+2) than that in 2000, with precipitation return periods of more than 100 and 10–20 years, respectively^27^. Also, the 2000 event occurred during the off-season for tourism in September whereas the exposure was much larger in 2015, because the event occurred in August when many tourists were present. Interruption of the peak holiday season caused severe indirect economic damage. Another example is a pair of droughts (ID 33) affecting North Carolina, United States. Between 2007 and 2009, about 65% of the state was affected by what was classified as an exceptional drought, with a composite drought indicator of the US Drought Monitor of 27 months^28^, whereas between 2000 and 2003 only about 30% of the state was affected by an exceptional drought of 24 months^28^. The crop losses in 2007–2009 were about €535 million, whereas they were €497 million in 2000–2003, even though vulnerability had been reduced due to drought early warning and management by the North Carolina Drought Management Council, established in 2003.

**Fig. 3:**
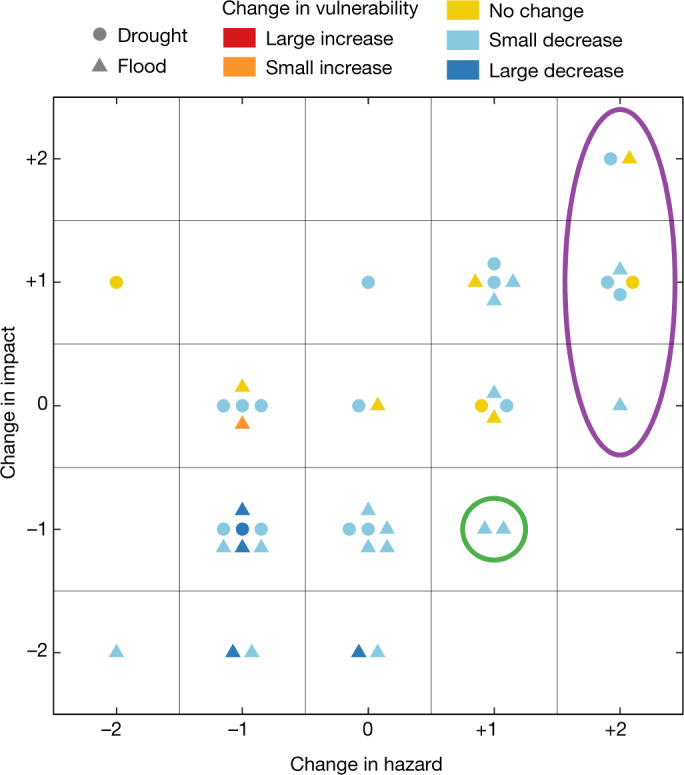
Relationship between change in hazard and change in impacts. Categories are: lower hazard and lower impact, ten cases; higher hazard and higher impact, 11 cases; lower hazard and higher impact, one case; higher hazard and lower impact, two cases. Circles and triangles indicate drought and flood paired events, respectively; their colours indicate change in vulnerability. Green circle highlights success stories (*n* = 2) of reduced impact (−1) despite a small increase in hazard (+1). Purple ellipse indicates paired events (*n* = 7) with large increase in hazard (+2)—that is, events that were subjectively unprecedented and probably not previously experienced by local residents. Source data

Box 1  Success stories of decreased impact despite increased hazard The dataset includes two cases in which a lower impact was achieved despite a larger hazard of the second event, making these interesting success stories (Fig. 3). Both cases are flood paired events, but of different types (that is, pluvial and riverine floods (Table 1)). These cases have in common that institutional changes and improved flood risk management governance were introduced and high investments in integrated management were undertaken, which led to an effective implementation of structural and non-structural measures, such as improved early warning and emergency response to complement structural measures such as levees (Table 1).

**Table 1:** Characteristics and commonalities in flood management of the two success stories.

	Pluvial floods in Barcelona, Spain (ID 12)		Riverine floods in Danube catchment in Germany and Austria (ID 15)	
Event characteristics	1995	2018	2002	2013
Hazard (hazard indicator-of-change +1)	Duration, 4 h; average event precipitation, 38 mm	Duration, 21 h; average event precipitation, 45 mm	7,700 m³ s^−1^ peak discharge at gauge Achleiten	10,100 m³ s^−1^ peak discharge at gauge Achleiten
Impacts (impact indicator-of-change −1)	€33.6 million^a^	€3.5 million	€4 billion^a^	€2.32 billion
**Commonalities in management changes: potential factors of success**				
Institutional changes, improved governance	Reorganization of early warning and emergency response after 1995, with improved collaboration between municipality, Catalonia and State Agency of Meteorology		Flood information service (HORA) for Austria went online in 2006; reorganization of flood warning and emergency response units with improved collaboration across federal states and transnationally	
High investments in structural and non-structural measures	About €136 million^a^ invested in structural measures alone, following the Integrated Sewerage Plan of Barcelona		Around €3.6 billion^a^ invested in flood risk management between events on structural and non-structural measures, including new legislation and building codes in Germany and Austria	
Strongly improved early warning and emergency response	New radar and lightning network plus operative mesoscale meteorological models in Catalonia, real-time control system based on rain gauge network and water level monitoring in Barcelona		Technical improvements in weather forecasting in Germany, much higher penetration rate of flood warnings and more effective flood response actions among citizens	

^a^Calculated as costs at the time of the second event.

## Effects of changes in management on drivers

The correlations shown in Fig. 2a,c also shed light on how management affects hazard, exposure and vulnerability and thus, indirectly, impact. For flood paired events, changes in management shortcomings are significantly positively correlated with changes in vulnerability (*r* = 0.56, *P* ≤ 0.01), and both are significantly positively correlated with changes in impact (Fig. 2a). For drought, however, these correlations are not significant (Fig. 2c). Thus, achieving decreases in vulnerability, and consequently in impact, by improving risk management (that is, reducing management shortcomings) seems to be more difficult for droughts than for floods. This difficulty may be related to spillover effects—that is, drought measures designed to reduce impacts in one sector can increase impacts in another. For example, irrigation to alleviate drought in agriculture may increase drought impacts on drinking water supply and ecology^29^.

The paired floods in the Piura region, Peru (ID 13) illustrate how effective management can reduce vulnerability, and consequently impact. At the Piura river, maximum flows of 3,367 and 2,755 m^3^ s^−1^ were recorded during the 1998 and 2017 events, respectively (that is, hazard showed a small decrease (−1)). Around 2000, the national hydrometeorological service started to issue medium-range weather forecasts that allowed preparations months before the 2017 event. In 2011, the National Institute of Civil Defence and the National Centre for the Estimation, Prevention, and Reduction of Disaster Risk were founded which, together with newly established short-range river flow forecasts, allowed more efficient emergency management of the more recent event. Additionally, non-governmental organizations such as Practical Action had implemented disaster risk-reduction activities, including evacuation exercises and awareness campaigns^30^. All of these improvements in management decreased vulnerability. The impact of the second event was smaller, with 366 fatalities in 1998 compared with 159 in 2017, despite an increase in exposure due to urbanization and population increase.

When the hazard of the second event was larger than that of the first (+1, +2), in 11 out of 18 cases (61%) the impact of the second event was also larger, irrespective of small decreases in vulnerability in eight of these cases (light blue dots/triangles in Fig. 3). There are only two paired events in our dataset for which a decrease in impact was achieved despite the second event being more hazardous (highlighted by the green circle in Fig. 3). These cases are considered success stories and are further discussed in Box 1. For the two paired events (ID 21 and 30) for which the only driver that changed was hazard (+1), the impacts did not change (0) (Extended Data Table 1). Water retention capacity of 189,881,000 m³ and good irrigation infrastructure with sprinkling machines were apparently sufficient to counteract the slight increase in hazard for the drought paired event in Poland in 2006 and 2015 (ID 21). The improved flood alleviation scheme implemented between the paired flood events (2016 and 2018), protected properties in Birmingham, United Kingdom (ID 30). There are, however, seven cases for which the second event was much more hazardous (+2) than the first (highlighted by the purple ellipse in Fig. 3)—that is, events of a magnitude that locals had probably not previously experienced. We term these events, subjectively, as unprecedented; almost all had an increased impact despite improvements in management.

One unprecedented pluvial flood is the 2014 event in the city of Malmö, Sweden (ID 45). This event was much more hazardous than that experienced a few years before, with precipitation return periods on average of 135 and 24 years, respectively, for 6 h duration^31^. The largest 6 h precipitation measured at one of nine stations during the 2014 event corresponded to a return period of 300 years. The combined sewage system present in the more densely populated areas of the city was overwhelmed, leading to extensive basement flooding in 2014 (ref. ^31^). The direct monetary damage was about €66 million as opposed to €6 million in the first event. An unprecedented drought occurred in the Cape Town metropolitan area of South Africa, in 2015–2018 (ID 44). The drought was much longer (4 years) than that experienced previously in 2003–2004 (2 years). Although the Berg River Dam had been added to the city’s water supply system in 2009, and local authorities had developed various strategies for managing water demands (for example, water restrictions, tariff increases, communication campaign), the second event caused a much higher direct impact of about €180 million^32^ because the water reserves were reduced to virtually zero.

Even though it is known that vulnerability reduction plays a key role in reducing risk, our paired-event cases reveal that when the hazard of the second event was higher than the first, a reduction in vulnerability alone was often not sufficient to reduce the impact of the second event to less than that of the first. Our analysis of drivers of impact change reveals the importance of reducing hazard, exposure and vulnerability to achieve an effective impact reduction (Fig. 2). Although previous studies have attributed a high priority to vulnerability reduction^17,21^, the importance of considering all three drivers identified here may reflect the sometimes limited efficiency of management decisions, resulting in unintended consequences. For example, levee construction aiming at reducing hazards may increase exposure through encouraging settlements in floodplains^33,34^. Similarly, construction of reservoirs to abate droughts may enhance exposure through encouraging agricultural development and thus increase water demand^35,36^.

Events that are much more hazardous than preceding events (termed unprecedented here) seem to be difficult to manage; in almost all the cases considered they led to increased impact (Fig. 3). This finding may be related to two factors. First, large infrastructure such as levees and water reservoirs play an important role in risk management. These structures usually have an upper design limit up to which they are effective but, once a threshold is exceeded, they become ineffective. For example, the unprecedented pluvial flood in 2014 in Malmö, Sweden (ID 45) exceeded the capacity of the sewer system^31^ and the unprecedented drought in Cape Town (ID 44) exceeded the storage water capacity^37^. This means that infrastructure is effective in preventing damage during events of a previously experienced magnitude, but often fails for unprecedented events. Non-structural measures, such as risk-aware land-use planning, precautionary measures and early warning, can help mitigate the consequences of water infrastructure failure in such situations^21^, but a residual risk will always remain. Second, risk management is usually implemented after large floods and droughts, whereas proactive strategies are rare. Part of the reason for this behaviour is a cognitive bias associated with the rarity and uniqueness of extremes, and the nature of human risk perception, which makes people attach a large subjective probability to those events they have personally experienced^38^.

On the other hand, two case studies were identified in which impact was reduced despite an increase in hazard (Box 1). An analysis of these case studies identifies three success factors: (1) effective governance of risk and emergency management, including transnational collaboration such as in the Danube case; (2) high investments in structural and non-structural measures; and (3) improved early warning and real-time control systems such as in the Barcelona case. We believe there is potential for more universal application of these success factors to counteract the current trend of increasing impacts associated with climate change^3^. These factors may also be effective in the management of unprecedented events, provided they are implemented proactively.

## Methods

The concept of paired events aims at comparing two events of the same hazard type that occurred in the same area^21^ to learn from the differences and similarities. This concept is analogous to paired catchment studies, which compare two neighbouring catchments with different vegetation in terms of their water yield^39^. Our study follows the theoretical risk framework that considers impact as a result of three risk components or drivers^3^: hazard, exposure and vulnerability (Extended Data Fig. 4). Hazard reflects the intensity of an event, such as a flooded area or drought deficit—for example, measured by the standardized precipitation index. Exposure reflects the number of people and assets in the area affected by the event. Consequently, the change in exposure between events is influenced by changes in the population density and the assets in the affected area (socioeconomic developments), as well as by changes in the size of the affected area (change of hazard). Vulnerability is a complex concept, with an extensive literature from different disciplines on how to define, measure and quantify it^13,40–42^. For instance, Weichselgartner^43^ lists more than 20 definitions of vulnerability, and frameworks differ quite substantially—for example, in terms of integration of exposure into vulnerability^11^ or separating them^3^. Reviews and attempts to converge on the various vulnerability concepts stress that vulnerability is dynamic and that assessments should be conducted for defined human–environment systems at particular places^12,44,45^. Every vulnerability analysis requires an approach adapted to its specific objectives and scales^46^. The paired event approach allows detailed context and place-based vulnerability assessments that are presented in the paired event reports, as well as comparisons across paired events based on the indicators-of-change. The selection of sub-indicators for the characterization of vulnerability is undertaken with a particular focus on temporal changes at the same place. All three drivers—hazard, exposure and vulnerability—can be reduced by risk-management measures. Hazard can be reduced by structural measures such as levees or reservoirs^19^, exposure by risk-aware regional planning^20^ and vulnerability by non-structural measures, such as early warning^21^.

Our comparative analysis is based on a novel dataset of 45 paired events from around the world, of which 26 event pairs are floods and 19 are droughts. The events occurred between 1947 and 2019, and the average period between the two events of a pair is 16 years. The number of paired events is sufficiently large to cover a broad range of hydroclimatic and socioeconomic settings around the world and allows differentiated, context-specific assessments on the basis of detailed in situ observations. Flood events include riverine, pluvial, groundwater and coastal floods^47–50^. Drought events include meteorological, soil moisture and hydrological (streamflow, groundwater) droughts^51^. The rationale for analysing floods and droughts together is based on their position at the two extremes of the same hydrological cycle, the similarity of some management strategies (for example, warning systems, water reservoir infrastructure), potential trade-offs in the operation of the same infrastructure^52^ and more general interactions between these two risks (for example, water supply to illegal settlements that may spur development and therefore flood risk). There may therefore be value in management communities learning from each other^18^.

The dataset comprises: (1) detailed review-style reports about the events and key processes between the events, such as changes in risk management (open access data; Data Availability statement); (2) a key data table that contains the data (qualitative and quantitative) characterizing the indicators for the paired events, extracted from individual reports (open access data); and (3) an overview table providing indicators-of-change between the first and second events (Supplementary Table 3). To minimize the elements of subjectivity and uncertainty in the analysis, we (1) used indicators-of-change as opposed to indicators of absolute values, (2) calculated indicators from a set of sub-indicators (Supplementary Table 1) and (3) implemented a quality assurance protocol. Commonly, more than one variable was assessed per sub-indicator (for example, flood discharges at more than one stream gauge, or extreme rainfall at several meteorological stations). A combination or selection of the variables was used based on hydrological reasoning on the most relevant piece of information. Special attention was paid to this step during the quality assurance process, drawing on the in-depth expertise on events of one or more of our co-authors. The assignment of values for the indicators-of-change, including quality assurance, was inspired by the Delphi Method^53^ that is built on structured discussion and consensus building among experts. The process was driven by a core group (H.K., A.F.V.L., K. Schröter, P.J.W. and G.D.B.) and was undertaken in the following steps: (1) on the basis of the detailed report, a core group member suggested values for all indicators-of-change for a paired event; (2) a second member of the core group reviewed these suggestions; in case of doubt, both core group members rechecked the paired event report and provided a joint suggestion; (3) all suggestions for the indicators-of-change for all paired events were discussed in the core group to improve consistency across paired events; (4) the suggested values of the indicators-of-change were reviewed by the authors of the paired-event report; and finally (5), the complete table of indicators-of-change (Supplementary Table 3) was reviewed by all authors to ensure consistency between paired events. Compound events were given special consideration, and the best possible attempt was made to isolate the direct effects of floods and droughts from those of concurrent phenomena on hazard, exposure and impact, based on expert knowledge of the events of one or more of the co-authors. For instance, in the course of this iterative process it became clear that fatalities during drought events were not caused by a lack of water, but by the concurrent heatwave. It was thus decided to omit the sub-indicator ‘fatalities’ in drought impact characterization. The potential biases introduced by compound events were further reduced by the use of the relative indicators-of-change between similar event types with similar importance of concurrent phenomena.

The indicator-of-change of impact is composed of the following sub-indicators: number of fatalities (for floods only), direct economic impact, indirect impact and intangible impact (Supplementary Table 1). Flood hazard is composed of the sub-indicators precipitation/weather severity, severity of flood, antecedent conditions (for pluvial and riverine floods only), as well as the following for coastal floods only: tidal level and storm surge. Drought hazard is composed of the duration and severity of drought. Exposure is composed of the two sub-indicators people/area/assets exposed and exposure hotspots. Vulnerability is composed of the four sub-indicators lack of awareness and precaution, lack of preparedness, imperfect official emergency/crisis management and imperfect coping capacity. Indicators-of-change, including sub-indicators, were designed such that consistently positive correlations with impact changes are expected (Supplementary Table 1). For instance, a decrease in 'lack of awareness' leads to a decrease in vulnerability and is thus expected to be positively correlated with a decrease in impacts. Management shortcomings are characterized by problems with water management infrastructure and non-structural risk management shortcomings, which means that non-structural measures were not optimally implemented. These sub-indicators were aggregated into indicators-of-change for impact, hazard, exposure, vulnerability and management shortcomings, to enable a consistent comparison between flood and drought paired events. This set of indicators is intended to be as complementary as possible, but overlaps are hard to avoid because of interactions between physical and socioeconomic processes that control flood and drought risk. Although the management shortcoming indicator is primarily related to the planned functioning of risk management measures, and hazard, exposure and vulnerability primarily reflect the concrete effects of measures during specific events, there is some overlap between the management shortcoming indicator and all three drivers. Supplementary Table 1 provides definitions and examples of description or measurement of sub-indicators for flood and drought paired events.

The changes are indicated by −2/2 for large decrease or increase, −1/1 for small decrease or increase and 0 for no change. In the case of quantitative comparisons (for example, precipitation intensities and monetary damage), a change of less than around 50% is usually treated as a small change and above approximately 50% as a large change, but always considering the specific measure and paired events. Supplementary Table 2 provides representative examples from flood and drought paired events showing how differences in quantitative variables and qualitative information between the two events of a pair correspond to the values of the sub-indicators, ranging from large decrease (−2) to large increase (+2). We assume that an event is unprecedented in a subjective way—that is, it has probably not been experienced before—if the second event of a pair is much more hazardous than the first (hazard indicator-of-change +2).

Spearman’s rank correlation coefficients are calculated for impact, drivers and management shortcomings, separated for flood and drought paired events. Despite the measures taken to minimize the subjectivity and uncertainty of indicator assignment, there will always be an element of subjectivity. To address this, we carried out a Monte Carlo analysis (1,000 iterations) to test the sensitivity of the results when randomly selecting 80% of flood and drought paired events. For each subsample correlation, coefficients and *P* values were calculated to obtain a total of 1,000 correlation and 1,000 *P* value matrices. The 25th and 75th quantiles of the correlation coefficients and *P* values were calculated separately (Extended Data Fig. 3).

## Online content

Any methods, additional references, Nature Research reporting summaries, source data, extended data, supplementary information, acknowledgements, peer review information; details of author contributions and competing interests; and statements of data and code availability are available at 10.1038/s41586-022-04917-5.

### Supplementary information

Supplementary Tables Supplementary Tables 1–3.

### Source data

Source Data Fig. 1.

Source Data Fig. 2.

Source Data Fig. 3.

**Extended Data Fig. 1:**
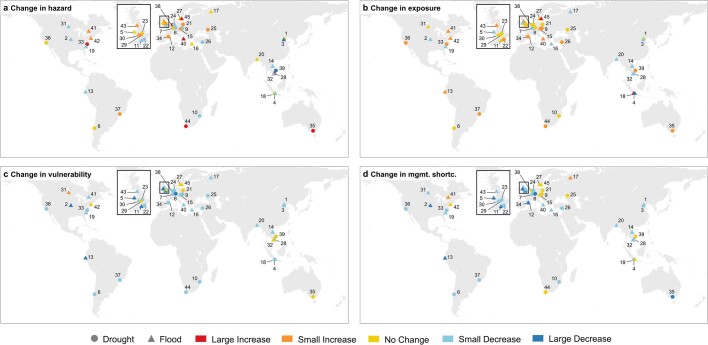
Location of flood and drought paired events coloured according to their indicators-of-change. **a**, Change in hazard; **b**, change in exposure; **c**, change in vulnerability and **d**, change in management shortcomings. Source data

Source Data Extended Data Fig. 1.

**Extended Data Fig. 2:**
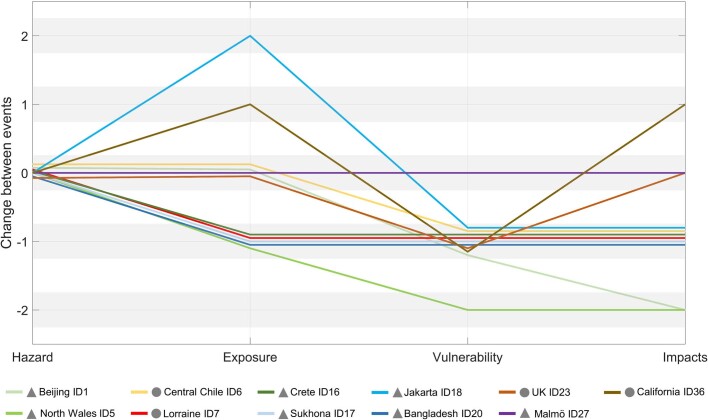
Parallel plot of paired events with the same hazard of both events. The hazard change is zero for all shown paired events. The lines show how the different combinations of indicators-of-change result in varying changes in impacts. Small offsets within the grey bars of the indicator-of-change values enable the visualization of all lines. Source data

Source Data Extended Data Fig. 2.

**Extended Data Fig. 3:**
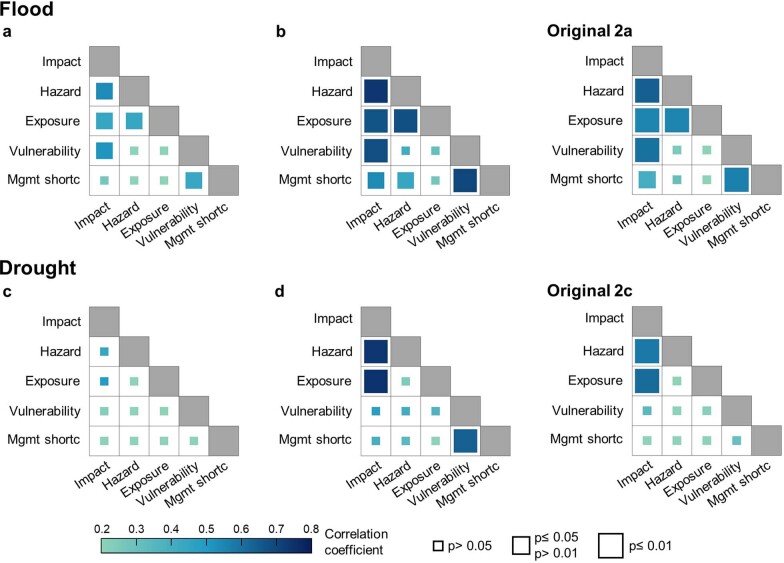
Results of the sensitivity analyses. **a–d** Correlation matrix of indicators-of-change for 25th and 75th quantiles of correlation coefficients and p-values, respectively (**a**, **c**) and 75th and 25th quantiles of correlation coefficients and p-values, respectively (**b**, **d**) separate for flood and drought paired events. Quantiles of correlation coefficients and p-values were calculated separately; colours of squares indicate Spearman’s rank correlation coefficients; sizes of squares indicates p-values. Fig. 2a, c is added to the right to ease comparison. Source data

**Extended Data Fig. 4:**
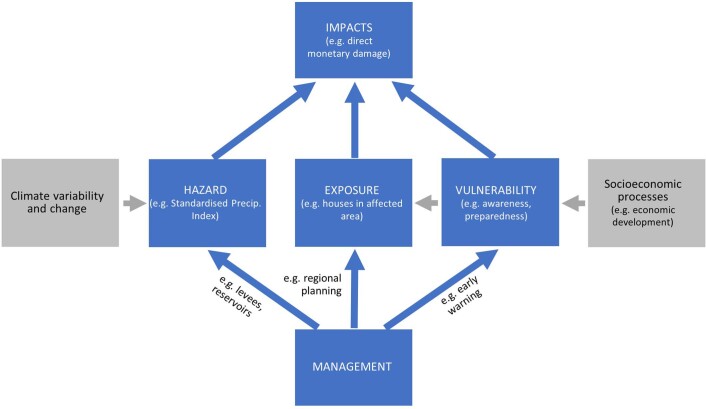
Theoretical framework used in this study (adapted from IPCC^3^). This theoretical risk framework considers impact as a result of three risk components or drivers: hazard, exposure and vulnerability, which in turn are modified by management.

Source Data Extended Data Fig. 3.

## Figures and Tables

**Extended Data Table 1:**
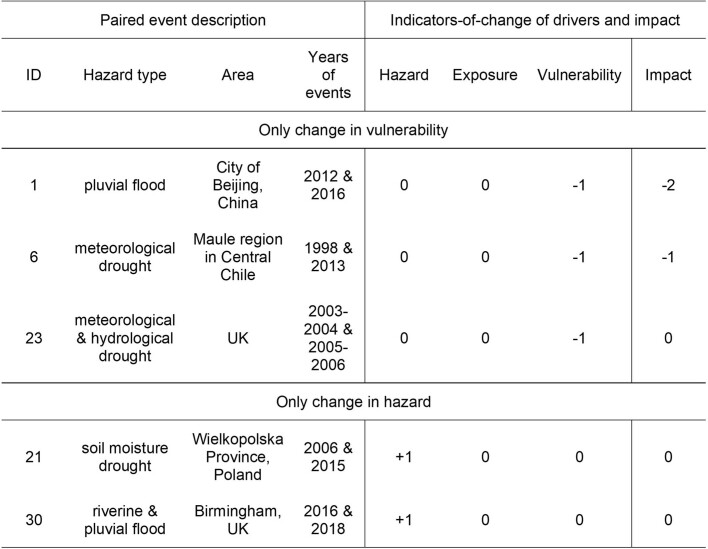
Overview of the indicators-of-change of paired events where only one of the three drivers has changed

## Data Availability

The dataset containing the individual paired event reports, the key data table and Supplementary Tables 1–3 are openly available via GFZ Data Services (10.5880/GFZ.4.4.2022.002). Source data are provided with this paper.
