# Supplementary material for: The challenge of unprecedented floods and droughts in risk management
Source: Nature. 2022 Aug 3;608(7921):80–6. doi: 10.1038/s41586-022-04917-5 (PMC9352573; doi:10.1038/s41586-022-04917-5)
Supplement: Supplementary file 1 — Supplementary Tables 1–3. [file 41586_2022_4917_MOESM1_ESM.pdf]

nature portfolio

https://doi.org/10.1038/s41586-022-04917-5

Supplementary information

The challenge of unprecedented floods and droughts in risk management

In the format provided by the
authors and unedited

Supplementary Material for

The challenge of unprecedented floods and droughts in risk management

H. Kreibich* et al.

* correspondence to Heidi Kreibich, GFZ German Research Centre for Geosciences, Section Hydrology, Telegrafenberg, D-14473 Potsdam, Germany, Phone: +49 (0)331/288-1550, Email: Heidi.Kreibich@gfz-potsdam.de

Contents:

- Supplementary Table 1: Definitions and examples of description or measurement of indicators characterising the impacts, the hazard, exposure and vulnerability as well as the management shortcomings for floods and droughts.
- Supplementary Table 2: Representative examples from flood and drought paired events of quantitative variables and textual descriptions corresponding to the five classes of change ranging from large decrease (-2) to large increase (+2) from the first event used as baseline to the second event of a pair. In case of quantitative comparisons, a change of less than 50% is treated as small, and above 50% as large.
- Supplementary Table 3: Indicators-of-change and sub-indicators indicate large change (-2/2), small change (-1/1) or no change (0) from the first event used as baseline to the second event of a pair.

Supplementary Table 1: Definitions and examples of description or measurement of indicators characterising the impacts, the hazard, exposure and vulnerability as well as the management shortcomings for floods and droughts.

Indicator	Definitions	Example description or measurement for floods	Example description or measurement for droughts
**Impact**			
Number of fatalities (only floods)	Number of fatalities due to the direct impact of a hazard.	Number of fatalities, e.g. reported in newspapers	*Not relevant*
Direct economic impacts	Direct economic impacts are due to the direct physical effect of a hazard on economic assets[14]	Flood damage to buildings expressed in Euros, e.g. recorded by insurance companies	Drought damage to crops expressed in Euros, e.g. quantified by compensation programmes
Indirect impacts	Indirect impacts occur inside or outside the hazard area, often with a time lag. They are commonly induced by direct impacts[14]	Disturbance of supply chains, e.g. described in economic reports	Loss of livelihoods, job loss in agriculture, e.g. described in governmental reports
Intangible impacts	Intangible impacts refer to damage to people, goods and services that are not easily measurable in monetary terms because they are not traded on a market (these can be direct or indirect impacts)[14]	Damage to cultural heritage, e.g. described by authorities	Damage to ecosystems, e.g. described by authorities
**Drivers of impact**			
**Hazard**			
Severity of flood/drought	Severity of the event in terms of hydro-meteorological processes, i.e. hazard	Maximum discharge measured at gauging station	Standardized Precipitation Evapotranspiration Index (SPEI), estimated based on the water balance
Duration of drought (only droughts)	Number of months in drought conditions[54]	*Not relevant*	Drought starts in the month when Standardized Precipitation Index falls below −1 and it ends when SPI returns to positive values
Precipitation / weather severity (only floods)	Heavy precipitation or severe weather that triggered the flood	Precipitation measured at weather stations	*Not relevant*
Antecedent conditions (only pluvial and riverine floods)	Conditions at the onset of an event that may exacerbate or mitigate the event[55]	Antecedent precipitation index, which is the weighted sum of past daily precipitation amounts, used as a proxy for soil moisture or: as an indicator for catchment wetness	*Not relevant*
Tidal level (only coastal floods)	Tidal water level at the time of coastal flood occurrence	Tidal water level measured at tide gauges	*Not relevant*
Storm surge (only coastal floods)	Rise in sea or estuary water level caused by the passage of a low pressure centre[55]	Sea water level measured at tide gauges	*Not relevant*
**Exposure**			
People/area/assets exposed	Number of people, size of area (e.g. settlement area, agricultural area) or number/value of assets located in affected areas[9]	Number of buildings in inundated area, e.g. estimated from satellite imagery	Number of inhabitants in drought affected area, e.g. from population statistics
Exposure hotspots	Areas of particularly high exposure affected during an event	Large scale industrial facility affected by flood	Hydraulic energy production affected by drought

**Vulnerability**			
Lack of awareness and precaution	Lack of understanding of the risk (e.g. sources, hazards, potential consequences, etc.) and implementation of suitable precautionary measures. Depends e.g. on experience, risk communication campaigns, incentives to implement precautionary measures	Ineffective risk communication, lack of guidelines and incentives for private precaution	Lack of drought experience
Lack of preparedness	Lack of knowledge and capacities developed by communities and individuals to effectively anticipate and respond to an event, e.g. via private emergency measures	Late early warning, insufficient resources like pumps, shutters, sandbags	Lack of water shortage response plans
Insufficient official emergency/crisis management	Organisational emergency or crisis management before or during an event was insufficient to optimally mitigate impacts	Lack of emergency plans, non-effective governance	Ineffective water demand management
Insufficient coping capacity	Coping capacity, which is the ability of communities using available skills and resources, to manage an event was insufficient due to a lack of funding (insurance, risk transfer), resources or skills	Low or lacking public flood compensation to individuals and businesses	Insufficient governmental aid or compensation
**Management shortcomings**			
Problems with water management infrastructure	Water management infrastructures such as levees, reservoirs, sewage systems, etc. failed or did not work optimally during an event due to deficits in maintenance, sub-optimal design, etc.	Number of levee breaches	Lack of water in reservoirs, insufficient storage capacity
Non-structural risk management shortcomings	Non-structural risk management measures, e.g. spatial planning that avoids increase of exposure in hazard-prone areas and private property level risk mitigation measures were not optimally implemented	Lack of hazard and risk maps	Ineffective water use restrictions

References

This are the additional references of Supplementary table 1 (for other references see reference lists of paper).

54. Spinoni, J. et al. World drought frequency, duration, and severity for 1951–2010. International Journal of Climatology, 34(8) 2792-2804 doi.org/10.1002/joc.3875 (2014)

55. WMO (World Meteorological Organization) & UNESCO (United Nations Educational, Scientific and Cultural Organization) International Glossary of Hydrology. WMO-No. 385 https://library.wmo.int/doc_num.php?explnum_id=8209 (2012).

Supplementary Table 2: Representative examples from flood and drought paired events of quantitative variables and textual descriptions corresponding to the five classes of change ranging from large decrease (-2) to large increase (+2) from the first event used as baseline to the second event of a pair. In case of quantitative comparisons, a change of less than 50% is treated as small, and above 50% as large.

**Impacts**					
**Impact indicators for floods**					
Indicators of change		**Number of fatalities**	**Direct economic impacts**	**Indirect impacts**	**Intangible impacts**
Large decrease (-2)	1st flood	Dead and missing: 4407 (ERD, 2008) (ID 20)	1,158 million USD (ERD, 2008) in 2007 values. Re-estimated as 1,329 million USD in the year 2009 and converted to 930 million EUR (ID 20)	Indirect damage of the flood event is estimated at USD 1,287 million for 2007 (Bappenas, 2007) (ID 4)	NA*
	2nd flood	Dead and missing: 190 (UNDP, 2010) (ID 20)	269.28 million USD (Xinhua, 2009) (converted to 188 million EUR) (ID 20)	Indirect damage of the flood event is estimated at USD 130 million for 2013 (Lurah Galur et al., 2013; Lurah Karet Tengsin et al., 2013; Lurah Petamburan et al., 2013) (ID 4)	NA
Small decrease (-1)	1st flood	9 fatalities (ID 15)	4 billion Euro (ID 15)	Some cascading effects due to damage to the gas network (ID 12)	Mercè festival events cancelled; damage to the Romanesque church of Sant Pere (ID 12)
	2nd flood	5 fatalities (ID 15)	2.32 billion Euro (ID 15)	no relevant indirect impacts (ID 12)	Damage to the Filmoteca (film library) and the Maritime Museum (ID 12)
No change (0)	1st flood	2 (indirect) fatalities in Saint-Anne-des-Monts, Quebec (IBC, 2019a; Peritz, Perreaux, & Stone, 2017) (ID 41)	[Total monetary damage unknown] CAD $223 million in insured damages (in 2017 value) (IBC, 2017). This is equivalent to CAD $230.06 million in 2019 value when adjusted for inflation (using Bank of Canada Inflation Calculator) (ID 41)	Common problems post-flooding include mould, contamination, debris. Other possible indirect economic impacts due to road closures; supply, use, and disposal of sandbags; costs associated with dispatching Canadian Armed Forces and supplies. However, specific numbers or problems have not been reported as of April, 2020	Water-borne diseases at informal residential areas along flooded canals in rainy seasons (HCM People's Committee, 2019; Huynh et al, 2020; Nguyen et al, 2017) (ID 28)

				(ID 41)	
	2nd flood	1 (indirect) fatality in Pontiac, Quebec (CBC, 2019a) (ID 41)	[Total monetary damage unknown] Insured losses reported to be CAD $208 million (in 2019 value) (IBC, 2019a). The estimate for financial assistance paid for 2019 flooding by Quebec is CAD $25.9 million as of June 2019 (Montreal Gazette, 2019) (ID 41)	Common problems post-flooding include mould, contamination, debris. Other possible indirect economic impacts due to road closures (Silcoff, 2019); supply, use, and disposal of sandbags; costs associated with dispatching Canadian Armed Forces and supplies. However, specific numbers or problems have not been reported as of April, 2020 (ID 41)	Water-borne diseases (Huynh et al, 2020; Nguyen et al, 2017) (ID 28)
Small increase (+1)	1st flood	0 fatalities (DRBC, 2006) (ID 42)	3.5 billion USD (at national level) (INDECI, 1998; CAF, 2000) (ID 13)	Comparatively small indirect loss due to the suspension of the tourist activities in the late holiday season in September, roads and railroads were temporarily interrupted (ID 40)	The Ontario portion of the Ottawa River was designated as a Canadian Heritage River in July 2016 to acknowledge its recreational and cultural value to Indigenous Peoples and its history as a transportation route (Government of Canada, 2016). The Ottawa River runs through the Algonquin Indigenous territories in Ontario that comprises ten Indigenous communities in Ontario (Water Canada, 2017). Flooding events along the river disrupt their traditional lifestyles and recreational activities (ID 41)
	2nd flood	4 fatalities (Suro et al., 2009) (ID 42=	3-9 billion USD (at national level) (Venkateswaran et al., 2017; INDECI, 2017) (ID 13)	High indirect loss due to the early suspension of the tourist activities at the peak of the holiday season in August, roads and railroads were temporarily interrupted (ID 40)	Similar disruptions as during the previous event due to flooding at the Ontario portion of the Ottawa River, a Canadian Heritage River (Government of Canada, 2016; Water Canada, 2017); Other long-term impacts comprise psychological impacts due to flooding fatigue caused by repeated flood events in similar regions or trauma due to emergency relocation and loss of belongings (Payne 2019, CBC, 2019b) (ID 41)
Large increase (+2)	1st flood	NA	SEK 60 million (GP, 2010) (ID 45)	NA	In post cyclone period, there was a rise in mental health related problems (Kabir et al., 2016). Sidr caused severe damage to the Sundarbans, which is a World heritage site (ERD,

					2008). However, the regeneration capacity of Sundarbans was high (Kumar Bhowmik and Cabral, 2013) (ID 20)
	2nd flood	NA	SEK 600 million in total; of this SEK 440 million paid by insurance (SOU 2017:42) (ID 45)	NA	A large number of people were displaced or migrated. In several areas, people could not return for 3-4 years due to continued tidal flooding. A large number of people changed their livelihoods to daily labor or fishing to cope (Kumar Paul, 2013; Abdullah et al., 2016). This change in livelihood had extreme impacts on their culture, standard of living and social status (ID 20)
**Impact indicators for droughts**					
			**Direct economic impacts**	**Indirect impacts**	**Intangible impacts**
Large decrease (-2)	1st drought		17,134 billion Euro (EEA, 2019a) (ID 9)	NA	NA
	2nd drought		2,172 billion Euro (EEA, 2019a) (ID 9)	NA	NA
Small decrease (-1)	1st drought		12% decrease in energy GDP regional contribution to the national energy GDP; 4% decrease in agriculture GDP regional contribution to the national agriculture GDP (computed as the difference between 1999 and 1998 GDP values from Banco Central de Chile, 2020) (ID 6)	Explosion of spruce and fir bark beetle (Geiger 1951) (ID 8)	Famine (Fegert, 2017), fish death (Deutscher Wetterdienst in der US-Zone 1947) (ID 8)
	2nd drought		13% increase in energy GDP regional contribution to the national energy GDP; 12% decrease in agriculture GDP regional contribution to the national agriculture GDP (computed as the difference between 2014 and 2013 GDP values from Banco Central de Chile, 2020) (ID 6)	Similar indirect impacts as in 1947 event, but easier to cope with. (ID 8)	Fish death (less than 1947) (ID 8)
No change (0)	1st drought		USD 50 million (EM-DAT (2019) (ID 10)	alga proliferation, 5% drop in electrical voltage, drought tax (ID 7)	Fish mortality and tree mortality (young plants) (ID 7)
	2nd drought		USD 70 million due to agricultural losses (Choudhary et al. 2015) (ID 10)	bar beetle epidemic, increase in climate multi-risk insurance (ID 7)	Significant and unusual tree mortality (Département de la santé des forêts, 2019) (ID 7)
Small	1st drought		10 to 12 billion US Dollars	Conflicts between different sectors of	Damage to the environment, soil

increase (+1)			(recalculated as at 2010) (ID 25)	water uses (hydraulic, tourism, irrigation, drinking water) (Ricart and Pavon, 2014) (ID 34)	erosion (Gibbs, 1984; Heathcote, 1988) (ID 35)
	2nd drought		15 billion US Dollars (ID 25)	Political conflicts between the party that was in the Government of Spain, the opposition and the Government of Catalonia, mainly because of the proposed transfer of water from Segre River to Internal Basins of Catalonia. Conflicts between hydroelectric, Water Catalan Agency, AGBAR for the overexploitation of water wells. (Llasat et al, 2009), newspaper La Vanguardia (2021) (ID 34)	Depression, exhaustion, drop in tourism, damaged aquatic and terrestrial ecosystems (Sherval et al., 2014, Bond et al. 2008; LeBlanc et al. 2012) (ID 35)
Large increase (+2)	1st drought		The estimated agricultural damage for 2003 is around 520,000 euros, the total agricultural damage is about 3% of the total crop value in the area. (ID 38)	Limited indirect impact (ID 44)	NA
	2nd drought		The estimated agricultural damage for 2018 is about 4 times as high as in 2003: 2,200,000 euros, which is about 11% of the total crop value in the area (ID 38)	About 35,000 job losses in agriculture, estimated 50,000 people pushed below poverty line due to job losses and food price inflation, drop in tourism (Ziervogel 2019; City of Cape Town 2019; WWF 2018) (ID 44)	NA
**Drivers of impact**					
**Hazard indicators for floods**					
		**Antecedent conditions**	**Precipitation/weather severity**	**Severity of flood**	
Large decrease (-2)	1st flood	Before the rains from Ivan arrived, the Delaware River at Montague and Trenton, New Jersey was flowing at 298 percent and 265 percent of normal, respectively, for the first half of September (DRBC, 2004, 2006) (ID 42)	Average precipitation in the southern part of basin was 595 mm; average precipitation in the northern part of basin was 410 mm (Wu 2006) (ID 3)	Total runoff of the southern part of basin was 5,995 billion m^3; total runoff of the northern part of basin was 1,539 billion m^3 (Wu 2006) (ID 3)	
	2nd flood	Normal to dry streamflow condition (Suro et al., 2009) (ID 42)	Areal mean rainfall in the north branch of Daqinghe river was 125 mm; areal mean rainfall in the south branch of Daqinghe river was 123 mm (Wu 2006) (ID 3)	Total volume into Baiyandian from north and south branch was 1,536 billion m^3 (Wu 2006) (ID 3)	
Small decrease (-1)	1st flood	Above-normal (150-200% of average) fall precipitation and saturated soils. High winter snowpack (90-130% of	327mm/6 days (Bappenas, 2007), 50 year RP (Bappenas, 2010) (ID 4)	Maximum recorded peak flow in Piura river ever (3367 m3 s-1) (ENFEN, 2017) (ID 13)	

		normal) with high snow water equivalent. Low winter temperatures and significant frost penetration (Manitoba Infrastructure, 2013; Blais et al. 2016) (ID 31)		
	2nd flood	Normal antecedent fall and winter conditions. Late spring melt and wet soils (Szeto et al. 2015; Ahmari et al. 2016) (ID 31)	250-300mm/15 days (Pertiwi, 2013), 30 year RP (Budiyono et al., 2016) (ID 4)	Peak flow of 2754.5 m3 s-1 (ID 13) (ENFEN, 2017)
No change (0)	1st flood	No rainfall in the last previous 3 days. Numerous inlets clogged by leaves (CLABSA, 1995) (ID 12)	Areal average April-May precipitation over the basin for period 1981-2010 was recorded to be 150 mm. In 2017, it was 257 mm (174% of average) (ORRPB, 2018). (ID 41)	4.16m surge (Adnan et al. 2019) plus low tide (ERD, 2008) (ID 20)
	2nd flood	No rainfall in the last previous 5 days. Some inlets clogged by leaves (BCASA, 2018) (ID 12)	April-May accumulated precipitation between 240-300 mm (preliminary data, Agriculture and Agri-Food Canada, n.d.) (ID 41)	4.10 m surge (Adnan et al. 2019) plus high tide (UNDP, 2010) (ID 20)
Small increase (+1)	1st flood	Late winter conditions and snowpack were considered average for the basin for May-April. Heavy localized rainfall events happening at the same time as snowmelt led to high soil saturation and river flows in early April. However, the primary driver of flooding was rainfall runoff (McNeil, 2019; ORRPB, 2018). (ID 41)	62.5 mm (Areal average of 3-day precipitation maxima for German part of the Upper Danube catchment) (Schröter et al., 2015) (ID 15)	7,700 m³/s peak discharge at gauge Achleiten (~HQ50) (HND 2021); 1,081 cm water level at gauge Passau; 10,250 m³/s peak discharge at Korneuburg/Vienna (Blöschl et al., 2013) (ID 15)
	2nd flood	Snow-cover did not reduce much till late April due to prolonged winter conditions. Snowpack/snow water equivalent in 2019 was considered to be 150-188% of average at peak amount. This led to increased freshet in late April. 2019 rainfall was above-average for the basin but less than that of 2017 and was more distributed over the basin. Hence, primary driver of flooding was a combination of above-average rainfall and snowmelt (McNeil, 2019 ; ORRPB, 2019).(ID 41)	75.7 mm (Areal average of 3-day precipitation maxima for German part of the Upper Danube catchment) (Schröter et al., 2015) (ID 15)	10,100 m³/s peak discharge at gauge Achleiten (~HQ150) (HND 2021); 1,289 cm water level at gauge Passau, i.e. highest water level in Passau since 1,501 flood; 11,055 m³/s peak discharge at Korneuburg/Vienna (Blöschl et al., 2013) (ID 15)
Large increase (+2)	1st flood	NA	Max precipitation: 175.26 mm, 50-to-100-year recurrence interval for a 24-	< 25 years return period of precipitation for 6-hour duration

			hour storm (Brooks, 2005) (ID 42)	(Sörensen & Mobini, 2017) (ID 45)	
	2nd flood	NA	Max Precipitation: 339.34 mm in 24 hours at Walton New York (Suro et al., 2009) (ID 42)	> 130 years return period of precipitation for 6-hour duration (Sörensen & Mobini, 2017) (ID 45)	
**Hazard indicators for droughts**					
			**Duration of drought**	**Severity of drought**	
Large decrease (-2)	1st drought		SPI6: 23 months, SPI12: 59 months (Cavus 2019; Cavus and Aksoy, 2019, 2020) (ID 26)	Average values for Maule region: SPI12 = -2.63; SPEI12 = -2.01 (ID 6)	
	2nd drought		SPI6: 9 months, SPI12: 13 months (Cavus 2019; Cavus and Aksoy, 2019, 2020) (ID 26)	Average values for Maule region: SPI12 = -0.95; SPEI12 = -1.06 (ID 6)	
Small decrease (-1)	1st drought		Hydrological drought duration: 3.4 years (ID 22)	The core of the 2003 drought event (12°W- 30°E; 35°N–55°N) recorded an extreme value of August SPEI3 = -1.62 (Schär et al., 2004) (ID 9)	
	2nd drought		Hydrological drought duration: 2.1 years (ID 22)	The core of the 2015 drought event (0°E- 45°E; 40°N–60°N) recorded an extreme value of August SPEI3 = -1.18 (Ionita et al., 2017) (ID 9)	
No change (0)	1st drought		May to September 2003, based on SPEI3 drought index (EDC, 2003a) (ID 9)	SPEI extremely dry (SPEI <-2) (ID 21)	
	2nd drought		Late May to September 2015 based on the SPEI3 drought index (Ionita et al., 2017) (ID 9)	SPEI extremely dry (SPEI <-2) (ID 21)	
Small increase (+1)	1st drought		24 months (NDMC 2020c ; NC DMAC 2020b) (ID 33)	Average inflow into reservoir system 57% lower than the long-term average (Araújo 1986) (ID 37)	
	2nd drought		27 months (NDMC 2020c ; NC DMAC 2020b) (ID 33)	Average inflow into reservoir system 77% lower than the long term average (Nobre et al. 2016) (ID 37)	
Large increase (+2)	1st drought		2 years annual rainfall below threshold (Jacobs et al. 2007) (ID 44)	At peak intensity, over 30% of area affected by exceptional drought (D4) (NDMC 2020b; NC DMAC 2020b) (ID 33)	
	2nd drought		4 years annual rainfall below threshold (Otto et al. 2018, Wolski 2018) (ID 44)	At peak intensity, over 60% of area affected by exceptional drought (D4) (NDMC 2020b; NC DMAC 2020b) (ID 33)	
**Exposure indicators for floods**					
		**People/area/assets exposed**	**Exposure hotspots**		

Large decrease (-2)	1st flood	More than 175,000 people exposed in South Carolina; at least 800,000 homes and businesses lost power access in South Carolina (Stewart, 2017) (ID 19)	NA
	2nd flood	About 40,000 people exposed in South Carolina; about 250,000 homes and businesses lost power access in South Carolina (Stewart, 2017) (ID 19)	NA
Small decrease (-1)	1st flood	8000 people and 4800 buildings exposed (Vologda regional government 2005) (ID 17)	50 flooded locations in the city (SCFC, 2011) (ID 28)
	2nd flood	7400 people and 2900 buildings exposed (Vologda regional government (2016) (ID 17)	31 flooded locations in the city, including the landing zone of Tan Son Nhat Airport (SCFC, 2016) (ID 28)
No change (0)	1st flood	Specifics around overall exposure of assets not well known (Westdal et al. 2015), but approximately similar between events. 3 million acres of cultivated farmland were exposed (MIT, 2013) (ID 31)	Flooding impacted primarily residential and city areas, including regions in Ontario (Dundas, Hamilton, Ottawa, Cumberland) and Quebec (Pontiac, Gatineau, Montreal island, Rigaud Saint-Jean sur Richelieu, Secteur Île Bizard, Île Mercier, Maniwaki, Mansfield-et-Pontrefact Shawinigan, Laval) (ORRPB, 2018; Floodlist, 2017) (ID 41)
	2nd flood	Specifics around overall exposure of assets not well known (Westdal et al. 2015), but approximately similar between events. About 2.5-3.5 million acres of cultivated farmland were exposed (AAFC, 2014) (ID 31)	Flooding impacted primarily residential and city areas, including regions in Ontario (Ottawa, Constance Bay, Fitzroy Harbour, Cumberland) and Quebec (Gatineau, Pontiac, Montreal, Sainte-Marthe-sur-le-Lac, Pointe-Calumet, Laurentians and the Chaudière Appalaches region) (Statistics Canada, 2019) (ID 41)
Small increase (+1)	1st flood	60,000 people exposed in Austria (EM-DAT, 2019) (ID 15)	Oldest part of the city, city center and cultural heritage (medieval walls and churches) exposed (ID 12)
	2nd flood	80,000 people exposed in Bavaria (likely not all of them in the Danube basin); 16697 residential houses in Bavaria and Baden-Württemberg exposed (likely not all of them in the	Oldest part of the city, city center, with great commercial and touristic activity and cultural heritage (medieval walls, churches, new Filmoteca (film museum and library))

		Danube basin) (Thieken et al., 2016a) (ID 15)	exposed (ID 12)
Large increase (+2)	1st flood	>350 buildings exposed, estimated on basis of flood claims to LF Skåne and (insurance company) and VA SYD (water utility company) (Sörensen & Mobini, 2017) (ID 45)	28 cities exposed, 2257 industrial, mining and railway enterprises in cities of Bao Ding, Xing Tai, Han Dan Shi Jia Zhuang and 116.4 km railway affected (Xiao et al. 1998) (ID 3)
	2nd flood	>4700 buildings exposed, estimated on basis of flood claims to LF Skåne and (insurance company) and VA SYD (water utility company) (Sörensen & Mobini, 2017) (ID 45)	91 cities exposed, 94,000 township enterprises, 15 national roads, 76 provincial roads and 396 bridges affected (Xiao et al. 1998) (ID 3)
**Exposure indicators for droughts**			
		**People/area/assets exposed**	**Exposure hotspots**
Large decrease (-2)	1st drought	NA	NA
	2nd drought	NA	NA
Small decrease (-1)	1st drought	Farmers across the UK exposed to soil moisture drought. No hosepipe bans so limited exposure to hydrological drought (Marsh, 2014; EA, 2017); Some local water supply difficulties in North West Scotland (Marsh, 2004) (ID 23)	In 1976 the drinking water supply was an exposure hotspot to drought especially in rural and industrial area because of insufficient drinking water network to satisfy the water demand (Mission interministérielle de l'eau 1977; Agence de l'Eau Rhin-Meuse 1977) (ID 7)
	2nd drought	Farmers in Eastern and Southern England exposed to soil moisture drought. Localised impact of hydro drought in the South and East of the UK (Marsh et al, 2014; EA, 2017) (ID 23)	Agricultural land (Chambre d'agriculture) and few rural villages exposed (decline of industry) (ID 7)
No change (0)	1st drought	Sown area: 1,488.2 thousand Ha; persons employed in agriculture: 205,275 (ID 21)	Drought hotspot at the Central Valley (urban and hydropower users) (ID 36)
	2nd drought	Sown area: 1,463.5 thousand Ha; persons employed in agriculture: 209,160 (ID 21)	Drought hotspot at the Central Valley (urban and hydropower users) (ID 36)
Small increase (+1)	1st drought	Large part of central Europe, ~3,700,000 km² (ID 9)	Cape Town domestic and industrial water users (Steenkamp, 2005) (ID 44)
	2nd drought	Whole Europe (Ionita et al., 2017), ~5,400,000 km² (ID 9)	Cape Town domestic and industrial water users & Western Cape Agricultural Users (Muller, 2018;

			WWF 2018) (ID 44)		
Large increase (+2)	1st drought	1,035,377 of inhabitants in the Adana province were exposed together with more from the Seyhan River basin (DPT, 2008) (ID 26)	NA		
	2nd drought	2,165,595 of inhabitants in the Adana province were exposed together with more from the Seyhan River basin (ID 26)	NA		
**Vulnerability indicators for floods**					
		**Lack of awareness and precaution**	**Lack of preparedness**	**Insufficient official emergency/crisis management**	**Insufficient coping capacity**
Large decrease (-2)	1st flood	Flood risk awareness of the population as well as authorities was limited and only few precautionary measures were undertaken before the event (ID 13)	The SENAMHI river flow forecasts and flood alerts did not yet exist for the 1998 event. Although weather forecasts existed, it can be assumed that these were much less precise than for the 2017 event (ID 13)	Official emergency management activities were limited (ID 13)	The capacity to manage localized flooding was significantly reduced in the early 1990s subsequent to the privatisation of the water industry in the UK (Pitt, 2007); household flood insurance was in place (during both flood events) (ID 11)
	2nd flood	NGOs such as 'Practical Action' have implemented disaster risk reduction activities such as evacuation exercises and awareness campaigns (French and Mechler; 2017); In 2011, the national Centre for the Estimation, Prevention, and Reduction of Disaster Risk (CENEPRED) was founded, which strongly improved risk awareness also among authorities (ID 13)	Around 2000, the national hydrometeorological service started issuing medium-range weather forecasts that allowed preparations months before the 2017 event. The national flood early warning system issued daily weather and river flow forecasts (SENAMHI, 2020) (ID 13)	The National Institute of Civil Defence (INDECI), and the national Centre for the Estimation, Prevention, and Reduction of Disaster Risk (CENEPRED), both founded in 2011, undertook and supported effective emergency management (ID 13)	Exposed communities formed networks and were able to effectively hold authorities to account. This means they were able to define their needs well and mobilise political support (e.g. the Pang Valley Flood Forum https://www.floodalleviation.uk/). This gave communities access to new funding for flood risk management, which requires evidence of effective local partnerships (ID 11)
Small decrease (-1)	1st flood	Last severe floods in 1974 and 1976. Prior to these floods, the 1954 Hurricane Hazel's flash-flooding resulted in 81 fatalities, which prompted Ontario to develop more stringent rules on infrastructure development on areas close to water (Perreaux, 2018) (ID 41)	Germany: penetration rate of early warning and actionable knowledge are low (Kreibich and Merz, 2007, DKKV, 2015, Kreibich et al. 2017) (ID 15)	In both, Germany and Austria, flood early warning was rather late and imprecise, coordination between the responsible authorities was limited (Thieken et al., 2016b, DKKV, 2015) (ID 15)	Economic compensations by state insurance "Consorcio de Compensación de Seguros" (CCS) helped to recover within several weeks (ID 12)
	2nd flood	Increased awareness since 2017 with more information available at various government and NGO websites on flood management and recovery (City	Penetration rate of early warning and actionable knowledge had increased significantly after 2002 event (Kreibich and Merz, 2007, DKKV,	In Germany and Austria: improved information and coordination capacities between the responsible authorities at federal, state and	Economic compensations by state insurance "Consorcio de Compensación de Seguros" (CCS) helped to recover within some days;

		of Ottawa, n.d.; Ottawa Riverkeeper, 2019; Pfeffer, 2019; Ontario Ministry of Natural Resource and Forestry, 2019; ORRPB, 2019) (ID 41)	2015, Kreibich et al. 2017) (ID 15)	community levels (Thieken et al., 2016b, DKKV, 2015) (ID 15)	the metro was fully operational again within a few hours (ID 12)
No change (0)	1st flood	Private precautionary measures implemented, such as storage of important items on higher level ground or upper floors of buildings, prepared door frames for shutters or dikes (Budiyono, 2018) (ID 4)	Happened Saturday evening, after rainfall all day (Sörensen & Mobini (2017); No official warnings or risk communication to the general public (ID 45)	Emergency management was supported by the military, as there were not enough emergency personnel available (MIT, 2013) (ID 31)	Main coping instruments include disaster recovery assistance (municipal, provincial, and federal when applicable) and private insurance (IBC, 2019b) (ID 41)
	2nd flood	Similar level of private precautionary measures implemented (Budiyono, 2018) (ID 4)	Happened early Sunday morning (4.30–7.30) when few people were in office, many people were sleeping (Sörensen & Mobini 2017); no official warnings or risk communication to the general public (Bentzel 2019) (ID 45)	Emergency management was supported by the military, as there were not enough emergency personnel available (Westdal et al. 2015) (ID 31)	Main coping instruments include disaster recovery assistance (municipal, provincial, and federal when applicable) and private insurance (McNeil, 2019) (ID 41)
Small increase (+1)	1st flood	High awareness and precaution - the Province recognized early in the fall of 2010 that there would be major flooding throughout Manitoba in the spring of 2011. Issued first spring flood outlook with high flood risk warning January 2011 (MIT, 2013). High knowledge and good operations of staff acknowledged as critical to successful management (MIT, 2013) (ID 31)	Manitoba Emergency Measures Organization began planning months ahead of flood event, including opening MB Emergency Coordination Centre (remained open for 103 days), purchasing 2 sandbag machines, etc. (MIT, 2013) (ID 31)	Responses to emergency calls were manageable (ID 45)	In 2011, Manitoba applied for Federal Disaster Financial Assistance Arrangements ($780 M) to help with recovery (Kavanagh and Annable, 2017), and also launched a $175 M compensation and mitigation program (Westdal et al. 2013) (ID 31)
	2nd flood	Less awareness and precaution because spring melt was complete and the flood did not resemble typical floods for the region (Healy, 2014). In 2014, the spring flood outlook predicted only minor to moderate risk (Ahmari et al. 2016) (ID 31)	In 2014, the Province had much less time to prepare for the flash flooding that occurred rather unexpected as it was a non-typical event for the basin (Healy, 2014) (ID 31)	Collaboration between different departments was good during the 2014 event, however a central coordinator would have been good since the roles and responsibilities were unclear. The warning was late and the staff were not mentally prepared for such an extreme event (Lindher, 2015) (ID 45)	In 2014, Manitoba applied for Federal Disaster Financial Assistance Arrangements ($180 M) to help with recovery (Kavanagh and Annable, 2017) (ID 31)
Large increase (+2)	1st flood	NA	NA	NA	NA
	2nd flood	NA	NA	NA	NA
**Vulnerability indicators for droughts**					
		**Lack of awareness and precaution**	**Lack of preparedness**	**Insufficient official emergency/crisis management**	**Insufficient coping capacity**
Large	1st drought	Low drought awareness, no	No warning systems, no seasonal	No special public management	No drought insurance available, the

decrease (-2)		precaution (ID 8)	forecast available for people and farmers (Hydrometeorological Center 1973, 1976) (ID 25)	organisation for droughts, no emergency plans available, volume of water redirected to Don irrigation system 2,5 km³ per year (ID 25)	food trade on the black market was a strategy to get food (Fegert, 2017) (ID 8)
	2nd drought	High drought awareness due to implemented monitoring systems and daily media reports (Erfurt et al. 2019) (ID 8)	Open-access 10-day and seasonal agro-meteorological forecast, warning system on Roshydromet website – MeteoAlarm service. For state water management company legislatively fixed critical water levels and early warning alarms when water levels are close to threshold (ID 25)	Public management organisation for droughts exists, drought emergency plans available, volume of water redirected to Don irrigation system 1,1 km³ per year, no watering of streets from June till September (ID 25)	In the case of a disaster on a national scale (like in the case of the drought 2018), the federal government of Germany provides financial assistance for forestry and agriculture (BMEL, 2019). Private insurances (yield guarantee insurances and damage-based insurances) exist for agriculture and forestry (BMEL, 2017) (ID 8)
Small decrease (-1)	1st drought	Mild awareness campaign to limit unnecessary water use (Jansen & Schulz 2006) (ID 44)	20% reduction in water allocation for domestic uses implemented by the City of Cape Town (Jacobs et al. 2007) (ID 44)	National and Local Water Demand Management; Level 3 or 4 Domestic Water Restriction in Cape Town Metropolitan Area up to 105 litres/per day (Jansen & Schulz 2006) (ID 44)	No insurance or governmental compensation (ID 7)
	2nd drought	Aggressive awareness campaign (Day Zero) to considerably reduce domestic and agricultural water consumption (Ziervogel 2019, Robins 2019, Rodina 2019) (ID 44)	Water use restrictions up to 60% for agriculture and 45% for domestic water (Ziervogel 2019, Robins 2019, Rodina 2019) (ID 44)	National, Local and International task force with emergency plan; Level 6 Domestic Water Restriction in Cape Town Metropolitan Area up to 50 litres/per day, Sanction, Tariff increase and Water Management Devices (Ziervogel 2019, Robins 2019, Rodina 2019) (ID 44)	Since 1982, law on compensation for victims of natural disasters (Law n°82-600, July 13, 1982). Farmers are advised to take private insurance (ID 7)
No change (0)	1st drought	High drought awareness in population (ID 6)	Early warning system did not exist (Aras et al., 2019) (ID 26)	No crisis management enacted (ID 23)	No drought insurance available (ID 37)
	2nd drought	High drought awareness in population (ID 6)	Early warning system did not exist (Aras et al., 2019), it is within the future program of public organizations. (ID 26)	No crisis management enacted (ID 23)	Insurance mechanisms proposed for hydrologic drought insurance under water demand and climate change scenarios in a Brazilian context (Mohor & Mendiondo, 2017), but not yet implemented (ID 37)
Small increase (+1)	1st drought	NA	NA	NA	Damage costs in agricultural and shipping sector mainly covered by higher prices: payed by consumer (Peters, 2003) (ID 38)
	2nd drought	NA	NA	NA	Resources in agricultural sector were not sufficient to cope with the consequences (Ecorys, 2019) (ID 38)
Large increase (+2)	1st drought	NA	NA	NA	NA

	2nd drought	NA	NA	NA	NA
**Management shortcomings**					
**Indicators of management shortcomings for floods**					
		**Problems with water management infrastructure**	**Insufficient risk management**		
Large decrease (-2)	1st flood	The design discharges of the levees were half the event discharges, all levees failed (Veatch, 1952) (ID 2)	Limited risk management activities and response capacity (French and Mechler 2017) (ID 13)		
	2nd flood	No levee failures occurred, following upgrading based on 1951 event (Lovelace & Strauser, 1996; United States General Accounting Office, 1995) (ID 2)	Much improved risk management and response capacity, including newly established government institutes (CENEPRED, INDECI) (French and Mechler 2017) (ID 13)		
Small decrease (-1)	1st flood	The combined capacity of the Portage Diversion (operated over design capacity during the flood event) and the dikes downstream of Portage La Prairie was not enough to contain peak flows, prompting the Province to construct an emergency controlled outlet at Hoop and Holler Bend (Blais et al. 2016; MIT, 2013) (ID 31)	No consistent large-scale flood hazard and risk mapping available before the event in 2002 (ID 15)		
	2nd flood	Directly following 2011 flood, an emergency outlet channel on the end of Lake St. Martin was constructed and operated over the winter to prepare for spring runoff. The operating rules for the Fairford Water Control Structure were also modified to allow maximum possible discharge to lower lakes levels between 2011-2014 (Ahmari et al. 2016); During the 2014 flood, the Portage Diversion was again operated over capacity (Ahmari et al. 2016). The emergency outlet at Hoop and Holler Bend was not required (ID 31)	Flood hazard mapping initiated following the EU Flood Directive launched in 2007; Floodplain restoration at lowland Danube tributaries in Germany and Austria since 2004 increased storage capacity (e.g. storage capacities at Salzach near Niedernsill, Austria) (BLFUW, 2006) (ID 15)		
No change (0)	1st flood	Issues with combined sewage system, Spillepengen pumping station out of order due to overload (Sörensen & Mobini, 2017) (ID 45)	Limited access to floodplain and flood risk maps (Henstra at al., 2019; Henstra & Thistlethwaite, 2018). Ontario guidelines for hydrologic modelling, floodproofing standards and floodplain mapping based on		

			approaches from the 1980s, now considered outdated (McNeil, 2019) (ID 41)
	2nd flood	Issues with combined and separate sewage system, Turbinen pumping out of order due to flooding (Sörensen & Mobini, 2017) (ID 45)	Still limited access to floodplain and flood risk maps (Henstra at al., 2019; Henstra & Thistlethwaite, 2018); Federal Floodplain mapping Framework containing guidelines for mapping projects released by government as part of National Disaster Mitigation Program (NRCan & Public Safety Canada, 2018). Government of Quebec announced CAD $24 million for updated flood zone maps after the 2017 event. Updated maps were released in June 2019, a month after the event (CTV Montreal, 2018; Anhoury, 2019). Federal Liberal government also earmarked CAD $2 billion to be spent over 11 years on risk mitigation and disaster prevention, but none of the approved projects were completed by 2019 floods (Press, 2017; Press, 2019a) (ID 41)
Small increase (+1)	1st flood	No dyke breaches (DKKV 2015) (ID 15)	NA
	2nd flood	Dyke failure along the Bavarian Danube and Isar resulted in extensive inundation at Deggendorf (24 km^2) (DKKV 2015) (ID 15)	NA
Large increase (+2)	1st flood	NA	NA
	2nd flood	NA	NA
**Indicators of management shortcomings for droughts**			
		**Problems with water management infrastructure**	**Insufficient risk management**
Large decrease (-2)	1st drought	System of reservoirs available to manage droughts (ID 35)	NA
	2nd drought	In 1984 the Thomson Reservoir was completed, which increased the existing storage capacity by 250% (Low et al. 2015) (ID 35)	NA

Small decrease (-1)	1st drought	The activation of stand-by sources and the granting of drought permits (EA 2004) to allow, for instance, additional abstraction to supplement dwindling reservoir stocks played an important role (Marsh, 2004) (ID 23)	Drought Monitoring Council Upgraded to Drought Management Advisory Council (NC DMAC 2020a) (ID 33)
	2nd drought	Some reservoirs were temporarily switched to non-consumptive mode (Marsh, 2007). Reduced water demand in 2006 meant that the major-pumped storage reservoirs for London were well sufficient (Marsh, 2007) (ID 23)	Requirement of local water providers to have Water Shortage Response Plans (North Carolina General Assembly 2007) (ID 33)
No change (0)	1st drought	Total retention capacity: 171,136 thousand m³; usable capacity of water reservoirs for melioration is 57,782 thousand m³ (ID 21)	Spray irrigation restrictions widely applied (Marsh, 2004). All the water companies in England and Wales revised their drought plans early in 2003 and the Environment Agency reported to Ministers on these in June 2003 (EA, 2004). Nearly all drought plans from companies were made public (EA, 2004). No hosepipe bans or restrictions on non- essential water use were applied (Marsh, 2004) (ID 23)
	2nd drought	Total retention capacity: 189,881 thousand m³; usable capacity of water reservoirs for melioration 53,878 thousand m³ (ID 21)	Spray irrigation restrictions widely applied. Introduction of a range of drought mitigation measures (e.g. publicity campaigns to moderate demand, local water transfers, reductions in compensation flows). Hosepipe bans, as well as appeals to save water, have been assessed by water companies to have reduced customers' demand for water by 5–15 per cent in 2006 (ID 23)
Small increase (+1)	1st drought	Well organised irrigation system (Hydrometeorological Center 1973, 1976; Dzhamalov et al. 2017) (ID 25)	NA
	2nd drought	Old and damaged irrigation system, no investment during last 30 years (Dzhamalov et al. 2017) (ID 25)	NA
Large	1st drought	NA	NA

increase (+2)			
	2nd drought	NA	NA

* NA – not such example available in dataset of paired events

Supplementary Table 3: Indicators-of-change and sub-indicators indicate large change (-2/2), small change (-1/1) or no change (0) from the first event used as baseline to the second event of a pair.

a Flood				*Management*			*Hazard*				*Exposure*			*Vulnerability*					*Impacts*				
Paired event ID	*Hazard type*	*Area*	*Years of events*	Problems with water management infrastructure	Non-structural risk management shortcomings	**Summary management shortcomings**	Antecedent conditions	Precipitation/weather severity	Severity of flood	**Summary hazard**	People/area/assets exposed	Exposure hotspots	**Summary exposure**	Lack of awareness and precaution	Lack of preparedness	Imperfect official emergency/crisis management	Imperfect coping capacity	**Summary vulnerability**	Number of fatalities	Direct economic impacts	Indirect impacts	Intangible impacts	**Summary impacts**
1	pluvial flood	City of Beijing, China	2012 & 2016	-1	NA	-1	0	0	0	0	0	0	0	-1	-1	-2	0	-1	-2	-2	-1	NA	-2
2	riverine flood	Kansas catchment, USA	1951 & 1993	-2	-2	-2	0	-1	-1	-1	-2	0	-1	-1	-2	-2	-1	-2	-2	-2	NA	NA	-2
3	riverine flood	Baiyangdian catchment, China	1963 & 1996	-1	-1	-1	1	-2	-2	-2	-2	2	-1	-1	-2	0	0	-1	-2	-1	NA	NA	-2
4	riverine flood	Jakarta, Indonesia	2007 & 2013	1	-1	0	0	-1	-2	-1	-2	NA	-2	0	-1	-1	NA	-1	-2	-2	-2	NA	-2
5	coastal flood	North Wales, UK	1990 & 2013	-1	-2	-2	NA	NA	0	0*	-2	0	-1	-2	-2	-2	NA	-2	0	-2	NA	NA	-2

11	groundwater flood	West Berkshire, UK	2000-2001 & 2013-2014	-1	-2	-2	-1	1	-1	-1	0	0	0	-1	-1	-2	-2	-2	0	-1	-1	0	-1
12	pluvial flood	Barcelona city, Spain	1995 & 2018	-2	-2	-2	0	0	1	1	1	1	1	0	-1	-2	-1	-1	0	-1	-1	-1	-1
13	riverine & pluvial flood	Piura region, Peru	1998 & 2017	NA	-2	-2	0	-2	-1	-1	1	1	1	-2	-2	-2	0	-2	-2	1	1	NA	-1
14	riverine flood	Mekong river, Cambodia	2000 & 2011	0	-1	-1	0	-2	0	-1	-1	0	-1	-1	-1	-1	0	-1	-1	0	0	1	-1
15	riverine flood	Danube catchment, Austria, Germany	2002 & 2013	1	-1	0	1	1	1	1	1	NA	1	-1	-1	-1	-1	-1	-1	-1	NA	NA	-1
16	riverine flood	Crete, Greece	1994 & 2015	-2	-1	-1	1	-1	0	0	-1	-1	-1	-1	-2	-1	-1	-1	0	-1	-1	0	-1
17	riverine flood	Sukhona catchment, Russia	1998 & 2016	1	0	1	-1	2	0	0	-1	0	-1	0	-1	-1	-1	-1	0	-2	NA	NA	-1
18	riverine flood	Jakarta, Indonesia	2002 & 2007	0	-1	-1	0	0	1	0	2	NA	2	-1	-1	-1	NA	-1	0	-2	-2	NA	-1
19	coastal flood	Charleston, USA	2016 & 2017	-1	-1	-1	NA	-1	-1	-1*	-2	0	-1	0	-1	0	0	-1	-1	-2	1	NA	-1
20	coastal flood	Coastal region of Bangladesh	2007 & 2009	-1	-1	-1	NA	-2	0	0*	-2	0	-1	-1	-1	-1	0	-1	-2	-2	NA	2	-1
27	pluvial flood	Malmö city, Sweden	2007 & 2010	0	NA	0	-1	NA	0	0	0	0	0	0	0	0	0	0	0	0	NA	NA	0

28	pluvial flood	Ho Chi Minh City, Vietnam	2010 & 2016	-1	-1	-1	0	2	2	2	0	-1	-1	-1	-1	0	-1	-1	0	1	-1	0	0
29	riverine & pluvial flood	Birmingham, UK	2008 & 2016	1	-1	-1	0	0	1	1	0	NA	0	-2	-1	-1	-1	-1	0	-1	0	0	0
30	riverine & pluvial flood	Birmingham, UK	2016 & 2018	-1	-1	-1	-1	0	1	1	0	NA	0	0	0	0	0	0	0	-1	0	0	0
31	riverine flood	Assiniboine catchment, Canada	2011 & 2014	-1	-1	-1	-1	1	-1	-1	0	0	0	1	1	0	1	1	0	0	NA	0	0
32	riverine, pluvial & coastal flood	Can Tho city, Hau river, Vietnam	2011 & 2016	0	-1	-1	-1	1	-1	-1	-2	0	-1	0	NA	NA	0	0	0	0	NA	NA	0
40	pluvial flood	Corigliano-Rossano city, Italy	2000 & 2015	-1	-1	-1	0	0	2	2	1	0	1	-1	-1	0	1	-1	0	1	1	NA	1
41	riverine flood	Ottawa river, Canada	2017 & 2019	1	0	1	1	0	1	1	1	0	1	-1	-1	-1	0	-1	0	0	0	1	1
42	riverine flood	Delaware catchment, USA	2004 & 2006	0	0	0	-2	2	1	1	1	1	1	-1	0	0	NA	0	1	1	NA	NA	1
43	riverine flood	Cumbria, UK	2009 & 2015	0	-1	-1	1	1	1	1	2	1	1	-1	0	-1	0	-1	0	1	0	NA	1
45	pluvial flood	Malmö city, Sweden	2010 & 2014	0	NA	0	0	NA	2	2	2	0	2	0	0	1	0	0	0	2	NA	NA	2

b Drought

Paired event ID	*Hazard type*	*Area*	*Years of events*	Problems with water management infrastructure	Non-structural risk management shortcomings	**Summary management shortcomings**		Duration of drought	Severity of drought	**Summary hazard**	People/area/assets exposed	Exposure hotspots	**Summary exposure**	Lack of awareness and precaution	Lack of preparedness	Imperfect official emergency/crisis management	Imperfect coping capacity	**Summary vulnerability**		Direct economic impacts	Indirect impacts	Intangible impacts	**Summary impacts**
6	meteorological drought	Maule region in Central Chile	1998 & 2013	NA	-1	-1		2	-2	0	1	-1	0	0	-1	-1	NA	-1		-1	NA	NA	-1
7	meteorological & hydrological drought	Lorraine region, France	1976 & 2018	-2	0	-1		-1	0	0	0	-1	-1	-1	-1	0	-1	-1		-1	0	0	-1
8	meteorological & hydrological drought	South-West Germany	1947 & 2018	-2	-1	-2		0	-1	-1	1	-1	0	-2	-1	-1	-2	-2		0	-1	-1	-1
9	meteorological drought	Central Europe	2003 & 2015	NA	0	0		0	-1	-1	1	0	1	-1	-1	-1	NA	-1		-2	0	-1	-1
10	hydrological drought	Limpopo catchment, Mozambique	1991 & 2005	NA	-1	-1		-1	-1	-1	0	0	0	NA	NA	-1	NA	-1		0	-1	NA	-1

21	soil moisture drought	Wielkopolska Province, Poland	2006 & 2015	0	0	0		1	0	1	0	0	0	NA	NA	NA	NA	0		0	0	NA	0
22	hydrological drought	Ver catchment, UK	2003-2006 & 2010-2012	0	-1	-1		-1	0	-1	1	0	1	-1	-1	-1	-1	-1		0	0	0	0
23	meteorological & hydrological drought	UK	2003-2004 & 2005-2006	-1	0	-1		2	-1	0	-1	1	0	-1	-1	0	-1	-1		0	0	0	0
24	hydrological drought	Meuse and Rhine catchments, EU	1976 & 2003	-1	-1	-1		-1	-1	-1	1	0	0	-1	-1	-1	NA	-1		0	0	NA	0
25	meteorological, soil moisture & hydrological drought	Don catchment, Russia	1972 & 2010	1	-1	0		1	0	1	1	-1	0	-1	-2	-2	-1	-1		1	0	0	0
26	meteorological drought	Seyhan River Basin, Turkey	1973 & 2014	-2	-1	-1		-2	0	-1	2	1	1	-1	0	-1	-1	-1		NA	NA	NA	0
33	meteorological, soil moisture & hydrological drought	North Carolina, USA	2000-2002 & 2007-2009	NA	-1	-1		1	2	2	1	NA	1	NA	-1	0	NA	-1		1	NA	NA	1
34	meteorological drought	Catalonia, Spain	1986-1989 & 2004-2008	0	-1	-1		1	1	1	1	1	1	-1	-1	-1	-1	-1		1	1	0	1

35	meteorological drought	Melbourne, Australia	1982-1983 & 2001-2009	-2	-1	-2		2	0	2	1	0	1	-1	0	0	NA	0		1	1	1	1
36	hydrological drought	California, USA	1987-1992 & 2012-2017	0	-1	-1		0	0	0	1	0	1	-1	-1	0	NA	-1		1	NA	1	1
37	hydrological drought	Sao Paulo, Brazil	1985-1986 & 2013-2015	-1	-1	-1		1	1	1	1	1	1	0	-1	-1	0	-1		NA	0	1	1
38	meteorological & hydrological drought	Raam catchment, The Netherlands	2003 & 2018-2019	0	-1	0		1	2	2	0	0	0	-1	-1	0	1	-1		2	NA	1	1
39	meteorological, soil moisture & hydrological drought	Central Highlands, Vietnam	2004-2005 & 2015-2016	-1	0	0		-2	-2	-2	1	0	1	0	0	-1	-1	0		2	1	0	1
44	meteorological drought	Cape Town area, South Africa	2003-2004 & 2015-2017	NA	0	0		2	2	2	1	1	1	-1	-1	-1	NA	-1		2	2	NA	2

* For coastal floods, additionally hazard sub-indicators tidal level (tl) and storm surge (ss) are determined as follows: ID 5: tl=0, ss=-1; ID 19: tl=+1, ss=-1, ID 20: tl=+1, ss=0
